# Functional outcomes of different surgical treatments for common peroneal nerve injuries: a retrospective comparative study

**DOI:** 10.1186/s12893-024-02354-x

**Published:** 2024-02-17

**Authors:** Zhen Pang, Shuai Zhu, Yun-Dong Shen, Yan-Qun Qiu, Yu-Qi Liu, Wen-Dong Xu, Hua-Wei Yin

**Affiliations:** 1 Department of Hand Surgery, Huashan Hospital, Fudan University, Shanghai, China; 2 Department of Hand and Upper Extremity Surgery, Jing’an District Central Hospital, Shanghai, China; 3 Department of Orthopedics and Hand Surgery, the First Affiliated Hospital of Fujian Medical University, Fujian, China; 4 Institute of Neuroscience, CAS Center for Excellence in Brain Science and Intelligence Technology, Chinese Academy of Sciences, Shanghai, China; 5 State Key Laboratory of Medical Neurobiology and MOE Frontiers Center for Brain Science, Institutes of Brain Science, Fudan University, Shanghai, China; 6 Priority Among Priorities of Shanghai Municipal Clinical Medicine Center, Shanghai, China; 7 The National Clinical Research Center for Aging and Medicine, Fudan University, Shanghai, China

**Keywords:** Common peroneal nerve injury (CPN injury), Neurolysis, Nerve repair, Tendon transfer

## Abstract

**Background:**

This study aims to assess the recovery patterns and factors influencing outcomes in patients with common peroneal nerve (CPN) injury.

**Methods:**

This retrospective study included 45 patients with CPN injuries treated between 2009 and 2019 in Jing’an District Central Hospital. The surgical interventions were categorized into three groups: neurolysis (group A; *n* = 34 patients), nerve repair (group B; *n* = 5 patients) and tendon transfer (group C; *n* = 6 patients). Preoperative and postoperative sensorimotor functions were evaluated using the British Medical Research Council grading system. The outcome of measures included the numeric rating scale, walking ability, numbness and satisfaction. Receiver operating characteristic (ROC) curve analysis was utilized to determine the optimal time interval between injury and surgery for predicting postoperative foot dorsiflexion function, toe dorsiflexion function, and sensory function.

**Results:**

Surgical interventions led to improvements in foot dorsiflexion strength in all patient groups, enabling most to regain independent walking ability. Group A (underwent neurolysis) had significant sensory function restoration (*P* < 0.001), and three patients in Group B (underwent nerve repair) had sensory improvements. ROC analysis revealed that the optimal time interval for achieving M3 foot dorsiflexion recovery was 9.5 months, with an area under the curve (AUC) of 0.871 (95% CI = 0.661–1.000, *P* = 0.040). For M4 foot dorsiflexion recovery, the optimal cut-off was 5.5 months, with an AUC of 0.785 (95% CI = 0.575–0.995, *P* = 0.020). When using M3 toe dorsiflexion recovery or S4 sensory function recovery as the gold standard, the optimal cut-off remained at 5.5 months, with AUCs of 0.768 (95% CI = 0.582–0.953, *P* = 0.025) and 0.853 (95% CI = 0.693–1.000, *P* = 0.001), respectively.

**Conclusions:**

Our study highlights the importance of early surgical intervention in CPN injury recovery, with optimal outcomes achieved when surgery is performed within 5.5 to 9.5 months post-injury. These findings provide guidance for clinicians in tailoring treatment plans to the specific characteristics and requirements of CPN injury patients.

## Background

Lower extremity nerve injuries represent 20% of all peripheral nerve injuries, among which the common peroneal nerve (CPN) is the most frequently damaged in the lower limb due to its superficial location [1, 2]. CPN injury often results in a “drop foot” symptom, with patients often exhibiting a characteristic steppage gait and suffering from ankle motor weakness in dorsiflexion [3]. The loss of great toe extension and dorsal foot sensory is also common [4]. The primary goal of surgical intervention is to enhance motor function, particularly in foot dorsiflexion, while also alleviating sensory disturbances and associated symptoms.

The choice of treatment for CPN injuries is heavily influenced by their underlying causes, which encompass various factors such as trauma, idiopathic entrapment, and iatrogenic injuries [5]. Traumatic etiologies include injuries such as lacerations, knee dislocations and fractures [6]. Idiopathic entrapment syndrome is the main cause of common peroneal palsies [7]. For instance, nerve lacerations necessitate immediate nerve repair, while neurolysis is suitable for addressing nerve entrapment. CPNs are frequently compressed by tendons, tumors or ganglion cysts, necessitating their resection during neurolysis procedures [8, 9]. Conventional treatment options include conservative management, physical therapy, neurolysis, nerve repair (comprising direct sutures and nerve grafting), and tendon transfer [10].

Considering that some common peroneal palsies may exhibit spontaneous recovery, non-operative management is usually preferred in cases lacking well-defined injuries [4]. Successful non-operative approaches include activity restriction and the utilization of ankle-foot orthoses. However, when functional improvement remains slow or absent despite 3–6 months of conservative therapy, surgical interventions become imperative [11]. Physical therapy techniques, such as electrical stimulation, have been found effective in promoting nerve repair and improving patient function [12].

Two primary surgical strategies are employed in the treatment of CPN injuries: (1) restoration of CPN function and (2) tendon transfer to reestablish foot muscle function and balance [13, 14]. Nerve exploration and neurolysis typically suffice for most entrapment or compression injuries, with 75% of patients demonstrating a positive nerve action potential during surgical exploration, achieving complete recovery [15]. In cases of sharp lacerations, direct nerve suturing within a few days is often the preferred choice. However, when the peroneal nerve exhibits defects or there is high anastomotic tension, autogenous nerve grafts are preferred, with the sural nerve serving as the most common donor. The success of nerve grafts is closely linked to graft length [16], as grafts shorter than 6 cm yield favorable outcomes in 64% of patients, while those exceeding 12 cm are associated with favorable outcomes in only 11% of patients [2]. In recent years, nerve transfer has emerged as a novel approach for CPN injury treatment. Transferring the soleus muscular branch of the tibial nerve to the deep fibular nerve has shown promise in CPN injury repair and the restoration of ankle dorsiflexion [17, 18]. Additionally, the double transfer of tibial nerve branches to the flexor digitorum longus and lateral head of the gastrocnemius to the deep peroneal nerve has proven beneficial in restoring motor function for certain patients [19]. These innovative approaches have opened new avenues in nerve repair therapy.

Neurolysis, being less invasive, facilitates rapid post-surgical recovery and effectively enhances the function of patients with intact CPN continuity. Nonetheless, its precise indications remain somewhat ambiguous. Neurolysis is less efficacious for patients with an unbroken CPN but experiencing complete motor function loss [20]. Nerve repair is appropriate for individuals with a complete CPN rupture, as it can restore both sensory and motor functions. However, when the graft length becomes excessive, nerve repair outcomes tend to be suboptimal.

Tendon transfer, particularly posterior tibial tendon transfer, is an effective method for reinstating foot dorsiflexion. The primary objective across all treatments remains the correction of foot drop, which can be achieved through tendon transfer [21]. Initially, tendon transfer was considered a corrective surgery for patients whose nerve function failed to improve after repair. However, a novel surgical approach has recently emerged, wherein tendon transfer is combined with nerve repair in a one-stage protocol, aimed at rebalancing muscle forces for enhanced reinnervation [22]. Ferraresi et al. have demonstrated that one-stage nerve repair and tendon transfer can yield superior functional recovery compared to nerve repair alone [23]. Similarly, Ho et al. reported that simultaneous tendon transfer and nerve repair may offer improved function compared to tendon transfer as a sole intervention [4].

Tendon transfer can effectively restore foot dorsiflexion but cannot fully restore muscle strength or range of motion and may result in flatfoot or hindfoot valgus [2, 20]. In addition, tendon transfer was less effective in restoring toe extension and dorsal foot sensory function.

While previous research has established the safety and efficacy of the three surgical treatments, there is a lack of studies investigating the postoperative recovery characteristics of each surgical approach, leading to a lack of evidence-based guidance for the decision-making process of physicians in selecting the most suitable surgery based on individual patient conditions and needs. Consequently, some patients may require a second surgery due to unsatisfactory results, particularly those who have previously undergone neurolysis. Thus, it is important to determine whether neurolysis can indeed yield the desired functional recovery.

Considering the limited number of systematic studies analyzing the factors that influence the prognosis of neurolysis, we designed this study to address these gaps by conducting a comprehensive retrospective analysis of patients post-treatment and investigating the factors that impact surgical outcomes. Our objective is to assess the therapeutic effects of different surgical interventions for CPN injuries and identify the key factors that influence the outcome of neurolysis to provide valuable guidance for clinical decision-making.

## Methods

### Study design and patient population

This descriptive, retrospective study included 45 patients with CPN injuries treated between 2009 and 2019 in Jing’an District Central Hospital. Patients were considered eligible if they met the following criteria: (1) confirmed CPN injury through examination, classified as partial or complete based on EMG grades, and attributable to diverse causes such as trauma, nerve entrapment, or idiopathic origins; (2) demonstrated weak foot dorsiflexion, graded as M0 to M4 on the Medical Research Council (MRC) scale for muscle strength, and/or sensory deficits on the dorsum of the foot; (3) underwent surgical treatments at Jing’an District Central Hospital, with comprehensive preoperative evaluation data available, and; (4) adhered to the follow-up recommendations. The study exclusion criteria were presence of severe organ dysfunction or severe ankle contracture/deformity on the affected side, inability to communicate normally due to severe neuropsychiatric disorders, and unwillingness of patients or their family members to participate in follow-up. The study was conducted in accordance with the ethical standards of the Declaration of Helsinki (as revised in 2013) and was approved by the Ethics Committee of Jing’an District Central Hospital (No. 202303), which waived the requirement for individual consent due to the retrospective nature of the present study.

### Treatment selection

The patients were classified into the following groups based on the treatments they underwent rather than the type of injuries; namely, Group A underwent neurolysis, Group B underwent nerve repair, and Group C underwent tendon transfer. The type of treatment was based on the surgeons’ discretions. Potential criteria for considering neurolysis were a closed injury or spontaneous compression in which preoperative electromyography (EMG) shows that stimulation proximal to the injury can elicit compound muscle action potentials at the target muscle, or although no signal is elicited on preoperative EMG, signals are re-recorded after intraoperative exploration of the nerve to release adhesions and open compression, and the texture of the injured nerve is good and continuity is still present; for nerve repair they were a direct cut injury in which the nerve is known to be ruptured preoperatively, or although the patient suffered a non-cut injury, intraoperative exploration reveals a neuroma-like structure at the site of the nerve injury and no evidence of nerve fiber regeneration on EMG; and for tendon transfer they were a patient who has been injured for more than a year and already has muscle atrophy such as a tibialis anterior, or who has had previous neurolysis or nerve repair surgery that has not been effective.

### Surgical technique

Patients received either lumbar anesthesia or general anesthesia while in the prone position, and the surgery followed standard sterile procedures with the application of a lower extremity tourniquet. The tourniquet was set at a pressure of 55 kPa and was in use for less than 60 min.

**Fig. 1:**
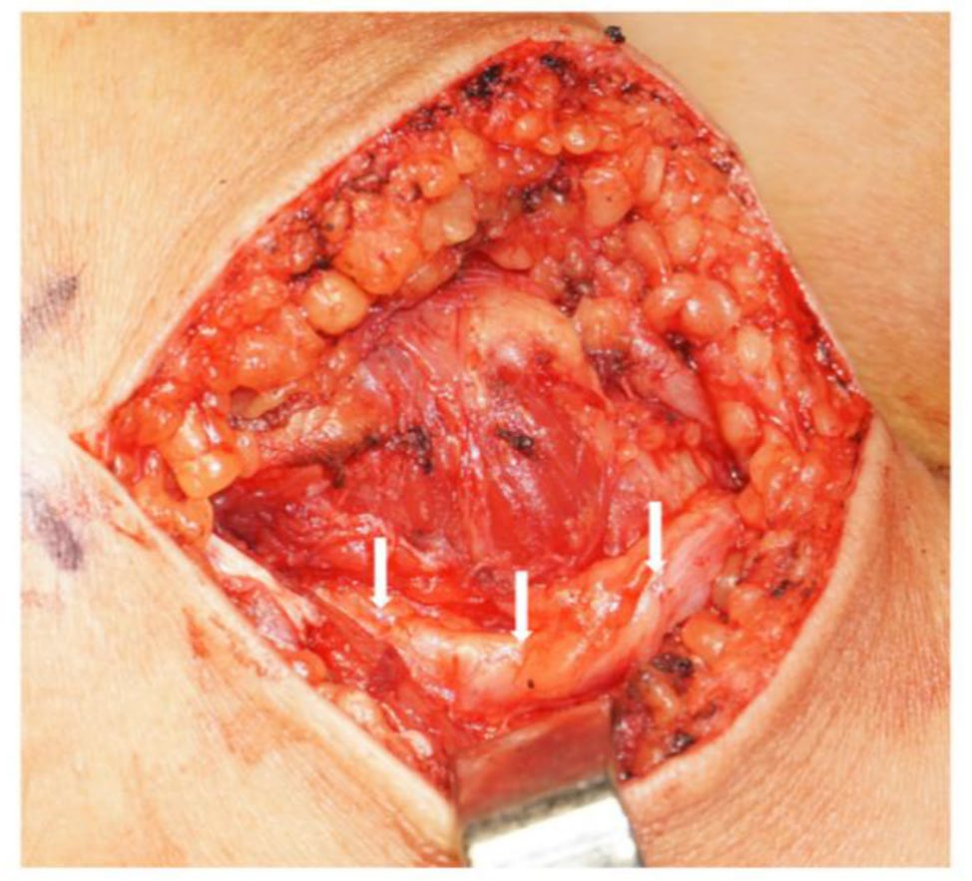
The release of the nerve distally. The white arrow indicates the common peroneal nerve after release. This is a lateral incision at the fibular head on the right leg, with the popliteal fossa on the right and the calf on the left

For neurolysis, a surgical oblique incision, typically 6–8 cm in length, was made extending from the fibular head to the popliteal space. The nerve, often entering the peroneal muscle layer near the head of the fibula, was tracked to locate the compression site. Frequently, the nerve is entrapped by the peroneus longus and brevis tendons or scar tissue. The tissue causing the nerve entrapment was excised, and in certain cases, partial dissection of the CPN’s epineurium was necessary (Fig. 1).

**Fig. 2:**
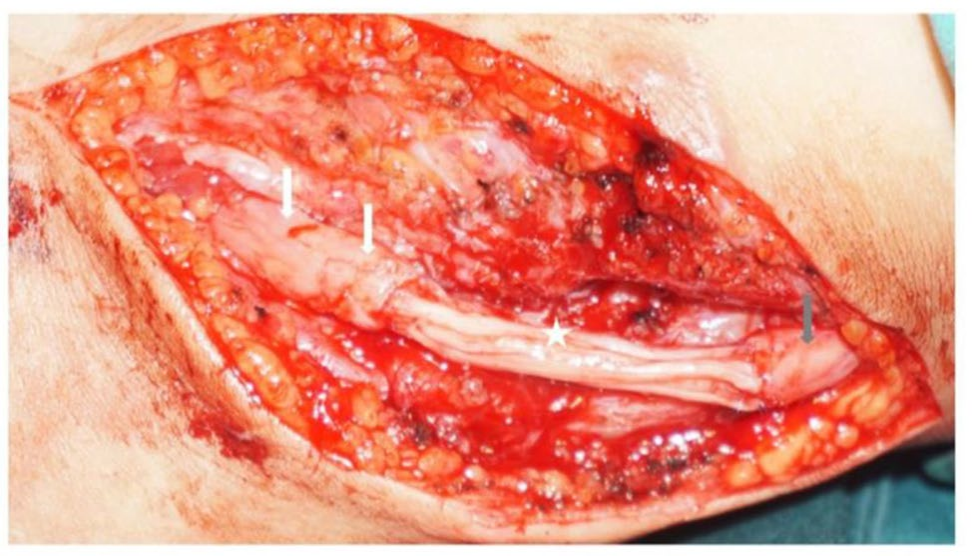
Suturing of the sural nerves between the common peroneal nerve stumps. The white arrow indicates the distal end of the common peroneal nerve, the gray arrow indicates the proximal end of the common peroneal nerve, and the white asterisk indicates the transplanted nerve

In the nerve graft procedure, an S-shaped incision of approximately 12 cm in length was made beneath the fibular head. The CPN was explored and released from the fibular head to the sites where the superficial and deep peroneal nerves bifurcate. Both nerves were separately exposed to confirm their continuity, and any ruptured or necrotic sections of the nerve were revealed. These damaged portions of the nerve were dissected, and the stumps on either side were trimmed to expose the healthy nerve papilla. The length of the nerve defect was then measured. When the defect gap was less than 1 cm, a direct suture was performed as the preferred approach. In cases with larger gaps, nerve grafting was required. The sural nerve, typically obtained through a surgical incision in the lateral calf, was used as the donor for nerve grafts, with the cut length of the sural nerve determined by the length of the CPN defect. Three- or four-strand sural nerves were employed in parallel to bridge the peroneal nerve (Fig. 2).

**Fig. 3:**
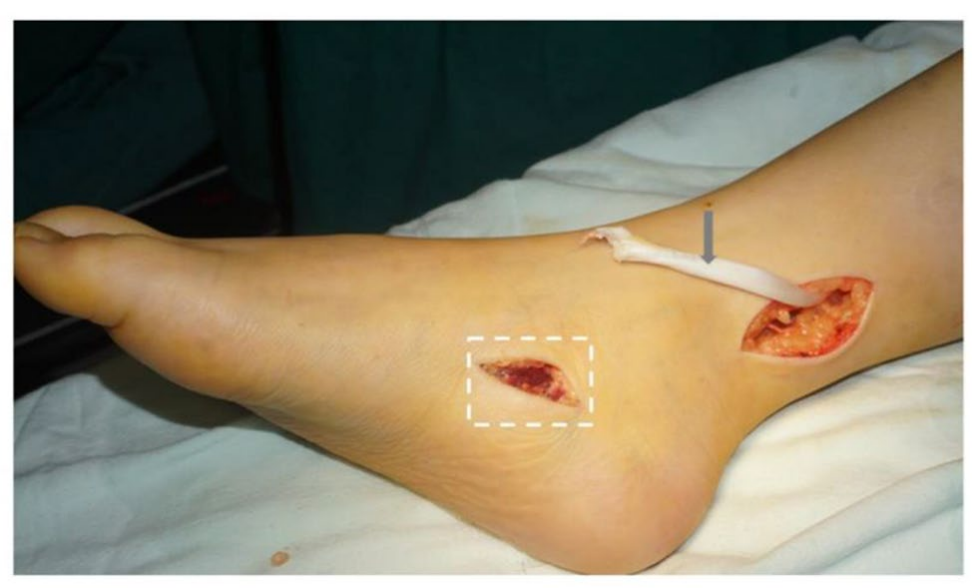
The posterior tibialis tendon was delivered from the wound on the medial calf. The gray arrow indicates the posterior tibialis tendon. The incision outlined by the white dotted line is used to find and resect the insertion of the posterior tibialis tendon. See Fig. 4 for the specific surgical operation

**Fig. 4:**
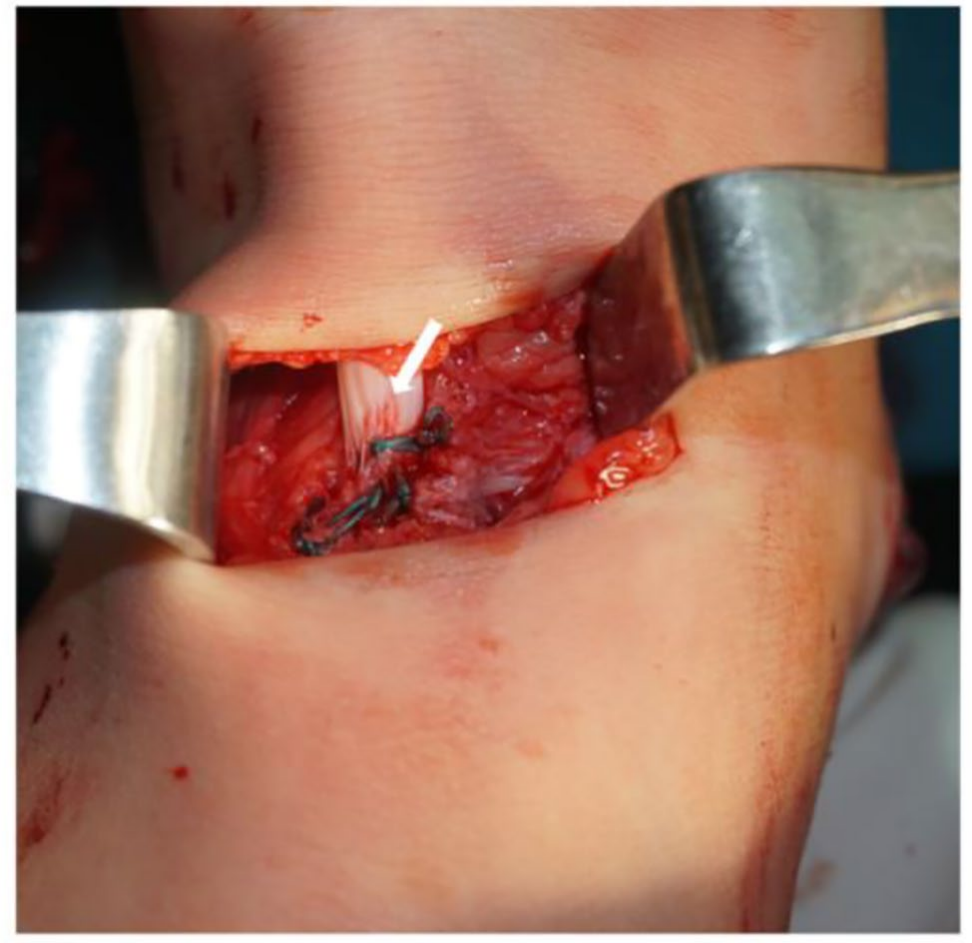
Fixing the tendon by sutures. The posterior tibialis tendon indicated by the white arrow is sutured to the third cuneiform bone. This is a front-and-rear view with the toe in front and the heel behind

In tendon transfer, two longitudinal incisions were made, one on the medial foot and the other on the medial calf. The posterior tibialis tendon was exposed and cut at its tendon insertion sites (Fig. 3). In some cases, the peroneal brevis tendon and flexor digitorum longus were also utilized for transfer. Subsequently, two longitudinal incisions were created, one over the dorsal surface of the foot and the other on the dorsal calf. An electric drill was used to perforate the diaphysis of the third cuneiform bone, extending through to the sole of the foot. The posterior tibialis tendon was guided through the tibiofibular interosseous membrane to reach the anterolateral foot incision. A puncture needle was used to facilitate the passage of the tendon through the cuneiform hole to the plantar surface, with the foot held at 80° of dorsiflexion. The site of tendon fixation was then sutured and reinforced (Fig. 4).

### Assessments

The following data were retrieved and assessed: demographic information, medical history, preoperative evaluations and postoperative outcomes. In both preoperative and follow-up physical examinations, motor strength and sensory function were assessed using the British Medical Research Council Scale. For motor rating comparison, we utilized the following convention: the standard S3 + sensory rating was designated as S4, and the standard S4 sensory rating was denoted as S5. Motor strength assessments were based on the anterior tibia-based foot dorsiflexion, soleus muscle-based foot plantarflexion, as well as toe dorsiflexion and plantarflexion. Sensory function scoring focused on the dorsal foot and lateral lower leg. Preoperative physical examinations also included Tinel’s sign evaluation at the fibular head. All patients underwent preoperative EMG to confirm the CPN injuries.

In the outcome evaluation, pain was assessed using the numeric rating scale (NRS) from 0 (no pain) to 10 (worst pain imaginable). The functional recovery level was assessed by questioning the patients on their activity level and participation in sports. The activity level included ambulatory walking, independent walking, and running. All of the patients were asked to rate their “overall satisfaction with the outcome of the operation” on a scale of extremely satisfied, satisfied, satisfied with reservation, and dissatisfied.

We considered a patient to have achieved good function if their motor grade was M3 or higher, while an M2 motor grade indicated fair function. Poor function was assigned for scores falling within the M0–1 range. Simultaneously, sensory functions are classified according to the same criteria as the above classification of motor functions [22].

### Follow-up

Follow-up was conducted through telephone, online communication software, or outpatient visits, with a minimum 1-year post-surgery duration and included assessing surgical efficacy (i.e., postoperative sensorimotor function, daily activities), detecting postoperative adverse events (i.e., pain, numbness), and evaluating satisfaction to the surgical treatments.

### Statistical analysis

Statistical analysis was performed using the SPSS V22 statistics tool (IBM Corporation, Armonk, New York, USA). Due to the nature of our data, non-parametric methods were predominantly used. Spearman correlation test assessed relationships between ordinal variables, such as the time interval from injury to surgery and postoperative functional outcomes. Graphs were generated using GraphPad Prism 7 software (Dotmatics, Boston, Massachusetts, USA). Data are presented as mean ± standard deviations. Quantitative variables were analyzed using Student’s *t*-test and qualitative variables using the Mann-Whitney U test.

Receiver operating characteristic (ROC) curve analysis was performed to determine the threshold time interval from injury to surgery for predicting postoperative foot dorsiflexion function, toe dorsiflexion function, and sensory function. To validate the predictive ability of the time elapsed from injury to surgery, the area under the curve (AUC) was computed, and the optimal cut-off points were identified based on the highest Youden Index.

Quantitative data were analyzed using the Student’s t-test for normally distributed variables and the Mann-Whitney U test for non-normally distributed variables. Subgroup analyses within Group A were performed using one-way analysis of variance (ANOVA) to identify factors associated with the outcome of neurolysis. Post-hoc tests incorporating Tukey correction were conducted to determine the significant differences among various subgroup means.

All comparisons were two-tailed, and statistical significance was determined based on *P* < 0.05.

## Results

### General clinical data of the patient

The study cohort included 35 males and 10 females, aged between 2 and 67 years old, with a mean age of 31.16 years old. Of them, 34 underwent neurolysis, five received nerve graft, and 6 underwent tendon transfer. On average, patients underwent neurolysis 6.1 ± 5.4 months (ranging from 0.5 to 24 months) after the onset of the disease, nerve graft 2.2 ± 0.4 months after disease onset (with four cases at 2 months and one case at 3 months), and tendon transfer 38.2 ± 23.3 months after disease onset (ranging from 10 to 72 months, with three cases under 36 months and three cases over 36 months). The patient groups were designated as groups A, B and C, corresponding to those who underwent neurolysis, nerve graft, and tendon transfer, respectively.

Regarding the nature of the injuries, ten patients experienced peroneal nerve injuries due to cut trauma (five of whom underwent neurolysis, as confirmed by electromyography and intraoperative exploration that revealed intact CPN continuity). Eight injuries resulted from traffic accidents, six from falls, six from knee dislocations, two from crush injuries, one from an unspecified trauma, eight from idiopathic nerve compression (including local compression and strenuous exercise), two from iatrogenic causes, one from poisoning, and one had an unknown cause (Table 1).

**Table 1:** Patient characteristics in the three assessed groups

Characteristics	Group A	Group B	Group C	Sum
**Number**	34	5	6	45
**Gender (male/female)**	28/6	5/0	2/4	35/10
**Age (years)**	33.91 ± 15.28	31.2 ± 14.66	15.5 ± 8.48	31.16 ± 15.76
**Myoatrophy (+/-)**	11/23	1/4	1/5	13/32
**Left/right**	19/15	2/3	2/4	23/22
**Injuries cause**				
**Trauma**	24	5	4	33
**Cut wounds**	5	4	1	10
**Traffic accident**	8	0	0	8
**Falling wound**	5	0	1	6
**Knee dislocation**	3	1	2	6
**Crush injury**	2	0	0	2
**Unknown**	1	0	0	1
**Local compression**	5	0	1	6
**Iatrogenic injury**	2	0	0	2
**Strenuous exercise**	2	0	0	2
**Poisoning**	1	0	0	1
**Unknown reason**	0	0	1	1

**Group A**: underwent neurolysis; **Group B**: underwent nerve graft; **Group C**: underwent tendon transfer

### Recovery of motor function

Before surgery, most patients had poor or fair foot dorsiflexion. However, nine patients in group A presented with good foot dorsiflexion function before the operation and their surgical indications primarily aimed to alleviate pain, further enhance functionality, improve toe dorsiflexion, and alleviate severe numbness.

The mean follow-up duration for the patients was 5.28 years. Compared to their preoperative levels, patients who underwent neurolysis (*P* < 0.001), nerve repair (*P* = 0.032) and tendon transfer (*P* = 0.015) all demonstrated improvements in foot dorsiflexion muscle strength after surgery. Specifically, in group A, 31 patients (91%) who received neurolysis achieved good foot dorsiflexion function, with 22 (71%) initially presenting with poor or fair dorsiflexion function preoperatively (Table 2). In group B, three patients (60%) who underwent nerve repair attained active dorsiflexion with a strength of M3. In group C, five patients (83%) who underwent tendon transfer achieved active dorsiflexion, demonstrating strengths ranging from M3 to M5 (Table 2). Notably, one patient in group B with fair recovery underwent secondary nerve repair. Furthermore, one patient in group C with poor recovery exhibited irreversible muscle atrophy. Toe dorsiflexion function, which is governed by the deep peroneal nerve branch of the CPN [24], was also monitored. In group A, 26 patients (76%) who underwent neurolysis achieved good toe dorsiflexion; in group B, three patients (60%) achieved good or fair toe dorsiflexion, with one patient in this group demonstrating an upgrade from fair to good function postoperatively; and in group C, one patient (17%) achieved good toe dorsiflexion (Table 3). Therefore, compared to nerve repair, tendon transfer proved more effective in restoring foot dorsiflexion function but displayed lower efficacy in restoring toe dorsiflexion.

**Table 2:** Results comparing the motor function (foot dorsiflexion) of the different groups

Group	Patients	Preoperative motor assessment			Postoperative motor assessment			Outcome			
		Good	Fair	Poor	Good	Fair	Poor	ES	S	SR	D
**A**	34	9	4	21	31	1	2	15	9	8	2
**B**	5	0	0	5	3	1	1	1	2	0	2
**C**	6	0	1	5	5	0	1	1	3	0	2

The motor grade is focused on foot dorsiflexion. ES, extremely satisfied; S, satisfied; SR, satisfied with reservation; D, dissatisfied. **Group A**: underwent neurolysis; **Group B**: underwent nerve graft; **Group C**: underwent tendon transfer

**Table 3:** Results comparing the motor function (toe dorsiflexion) of the different groups

Group	Patients	Preoperative motor assessment			Postoperative motor assessment		
		Good	Fair	Poor	Good	Fair	Poor
**A**	34	1	5	22	26	1	7
**B**	5	0	1	4	2	1	2
**C**	6	1	0	4	1	0	5

The motor grade is focused on toe dorsiflexion. 6 patients with neurolysis in Group A and 1 patient in group C had no records of preoperative toe dorsiflexion. **Group A**: underwent neurolysis; **Group B**: underwent nerve graft; **Group C**: underwent tendon transfer

In group A, patient satisfaction levels were as follows: 15 patients (44%) were extremely satisfied, nine patients (26%) were satisfied, eight patients (24%) were satisfied with reservation, and two patients (6%) were dissatisfied. In group B, one patient (20%) was extremely satisfied, two patients (40%) were satisfied, and two patients (40%) were dissatisfied. In group C, one patient (17%) was extremely satisfied, three patients (50%) were satisfied, and two patients (33%) were dissatisfied (Table 2). Patients in groups B and C who had fair or poor dorsiflexion outcomes expressed dissatisfaction. Additionally, one patient in group C, a 4-year-old, expressed dissatisfaction as the patient had hoped for more substantial improvements in foot dorsiflexion and toe dorsiflexion.

### Recovery of sensory function

Neurolysis effectively restored sensory function in the dorsal foot and lateral lower leg for patients with CPN injuries (*P* < 0.001). However, patients who underwent nerve repair (*P* = 0.310) or tendon transfer (*P* = 0.699) did not show significant improvements in sensory function after surgery compared to their preoperative status. In group A, 30 patients (88%) experienced substantial sensory function recovery in the dorsal foot and lateral calf (Table 4). In group B, two patients had improved their sensory function from poor to fair, and one patient from fair to good, while the remaining patients exhibited no changes in sensory function. In Group C, one patient’s sensory function in the dorsal foot and lateral calf improved from fair to good. Comparatively, nerve repair appeared more effective than tendon transfer in restoring sensory function.

**Table 4:** Results comparing the sensory function and motion of the different groups

Group	Patients	Preoperative sensory assessment			Postoperative sensory assessment			Numbness	Activity		
		Good	Fair	Poor	Good	Fair	Poor		WA	Walk	Run
**A**	34	4	16	12	30	3	1	17	0	11	23
**B**	5	1	2	2	2	3	0	4	0	3	2
**C**	6	1	5	0	2	4	0	1	1	2	3

The preoperative sensory function evaluation data was lost in two patients in group A. The sensory grade and numbness were focused on the dorsal foot and lateral lower leg. WA, walks with ambulatory aids. **Group A**: underwent neurolysis; **Group B**: underwent nerve graft; **Group C**: underwent tendon transfer

Among the patients, numbness in the dorsal foot and lateral calf was reported by 17 patients (50%) in group A. In contrast, four patients (80%) in group B and only one patient (17%) in group C reported numbness. In terms of the highest level of achieved activity, 23 patients (68%) in group A were able to run, and 11 patients (32%) could walk unaided. In group B, two patients (40%) could run, while three patients (60%) were limited to walking. In group C, three patients could run, and two patients could walk barefoot after tendon transfer. Pain was infrequently reported among these patients, with only three patients (9%) in group A describing slight pain. Among the five patients in group B, one reported severe pain with an NRS rating of 7. In group C, two patients (33%) experienced pain, with ratings of 3 or 5. All patients underwent Tinel’s sign testing at the lateral aspect of the fibular head and neck. Most of the 34 patients in the neurolysis group, two of the five patients in the nerve repair group, and four of the six patients in the tendon transfer group exhibited positive results. However, no significant relationship was observed between the presence of Tinel’s sign and surgical outcomes.

### Factors affecting the prognosis of neurolysis

Three neurolysis patients later received tendon transfer to improve motor function. For individuals with suboptimal neurolysis outcomes, alternative surgeries were considered to enhance functional recovery. Factors influencing neurolysis outcomes were then explored, and the results indicated that foot dorsiflexion recovery showed no significant age correlation (ρ = 0.052, *P* = 0.77). However, it exhibited a weak correlation with preoperative EMG results (ρ = 0.353, *P* = 0.04) and a significant negative correlation with the time from onset to surgery (ρ=−0.481, *P* = 0.004). Patients with preoperative EMG findings suggesting partial CPN injury tended to achieve better neurolysis outcomes than those with complete CPN injury. Moreover, shorter intervals between onset and surgery were associated with improved neurolysis results.

**Fig. 5:**
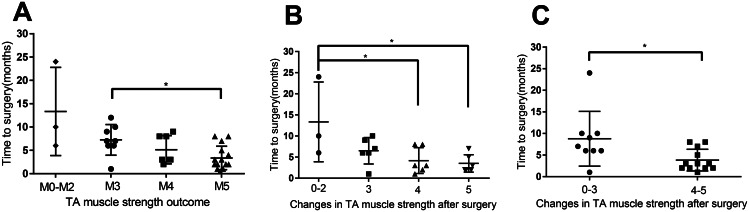
The tibialis anterior muscle force among the three groups. **A**. Relationship between the time from symptom onset to neurolysis and post-surgery muscle strength (M3 vs. M5). Patients with M5 muscle strength after surgery (3.37 ± 2.43) had a significantly shorter period from symptom onset to surgical treatment compared to those with M3 muscle strength (7.25 ± 3.07). *P*=0.011. Data from one patient, whose muscle strength had recovered to M5, was excluded from the analysis due to an unusually long time interval (24 months) between symptom onset and neurolysis. When including this patient’s data, the time interval for patients with M5 was 4.66 ± 5.52, and the *P* value was 0.0549. **B-C**. Duration from symptom onset to neurolysis in relation to changes in tibialis anterior muscle force. B. Patients with 4 (4.16 ± 2.79) or 5 (3.5 ± 1.89) grade muscle strength improvements exhibited shorter time intervals than those with 0–2 improvements (13.33 ± 7.72). *P*=0.03 (compared to 4 grade improvements), *P*=0.02 (compared to 5 grade improvements). **C**. Patients with 4-5 grade muscle strength improvements (3.83 ± 2.41) had shorter time intervals than those with 0–3 improvements (8.78 ± 5.98). *P*=0.03. *, *P*<0.05; error bars represent the standard deviation (SD). *n*=33 for panel **A**, *n*=21 for panels **B-C**. TA, tibialis anterior

When assessing motor function recovery, we focused on the tibialis anterior muscle responsible for foot dorsiflexion, a crucial aspect impacting patients’ daily lives. A correlation was observed between higher postoperative foot dorsiflexion muscle strength and shorter time intervals (Fig. 5A). However, due to variations in preoperative muscle strengths among patients, the reliability of this finding is limited. Subsequently, we narrowed our analysis to 21 patients with poor preoperative dorsiflexion muscle strength (Table 2) to investigate changes in muscle strength after neurolysis. Consistent with previous results, shorter time intervals were associated with greater functional improvements (Fig. 5B-C). However, statistical significance in Fig. 5B is limited due to the small number of patients with muscle strength changes from 0 to 2 (*n* = 3). Combining patients with 0–2 grade changes and those with 3 grade changes into one group, we found that a short time from symptom onset to surgery can lead to substantial foot dorsiflexion functional recovery (muscle strength increased by 4–5). However, a longer interval did not necessarily imply a lack of functional recovery, as muscle strength can still improve by 0–3.

**Fig. 6:**
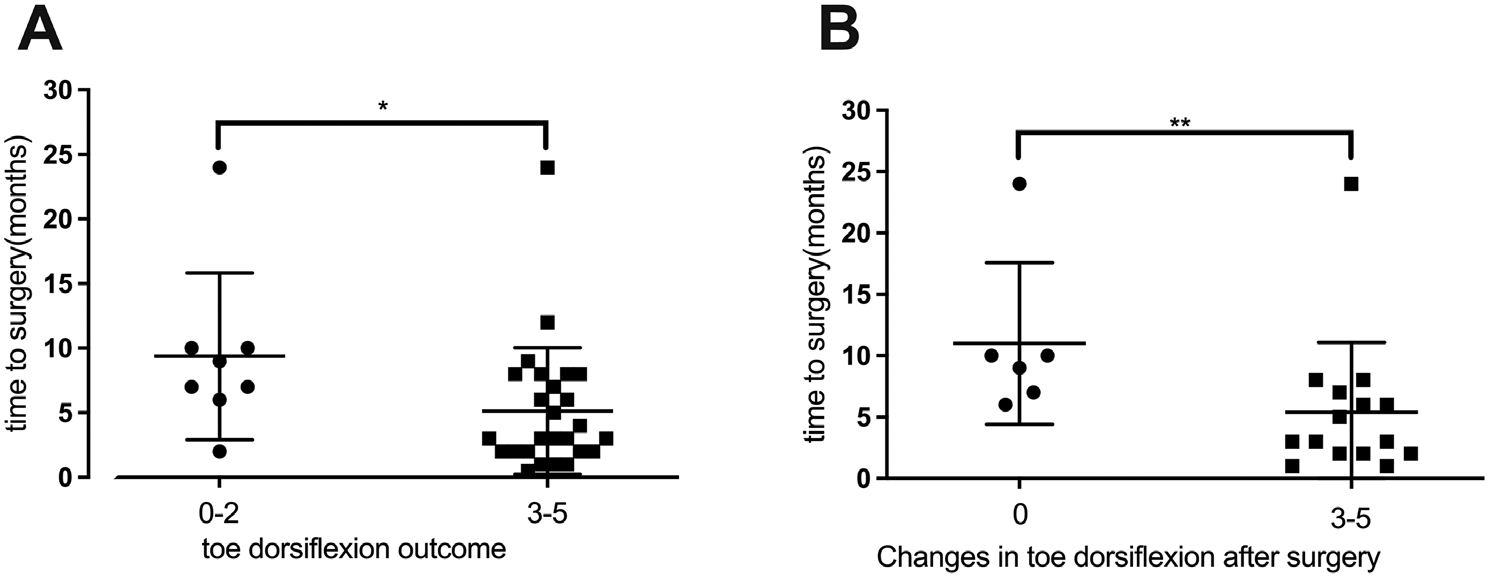
The toe dorsiflexion muscle strength among the three groups. **A**. Relationship between the time from symptom onset to neurolysis and post-surgery toe dorsiflexion muscle strength (M0-M2 vs. M3-M5). Patients with M3 to M5 muscle strength (5.14 ± 3.06) had significantly shorter time intervals between symptom onset and surgical treatment compared to patients with M0 to M2 muscle strength (9.38 ± 5.74). *P*=0.04. **B**. Graph illustrating that patients with 3 to 5 grade muscle strength improvements had shorter time intervals (5.4 ± 5.5) than those without improvements (11 ± 6). *P*=0.0074. *, *P*<0.05; **, *P*<0.01; error bars represent the standard deviation (SD). *n*=34 for panel **A**, *n*=21 for panel **B**

Investigation of toe dorsiflexion function recovery showed a significant association between better recovery and shorter time intervals (Fig. 6A). Analysis of patients who lacked toe dorsiflexion before surgery (*n* = 22 patients; Table 3) showed that a shorter time interval between symptom onset and surgery correlated with improved toe dorsiflexion (Fig. 6B). It was found that once a specific time threshold was exceeded, patients lost the opportunity to restore toe dorsiflexion function.

**Fig. 7:**
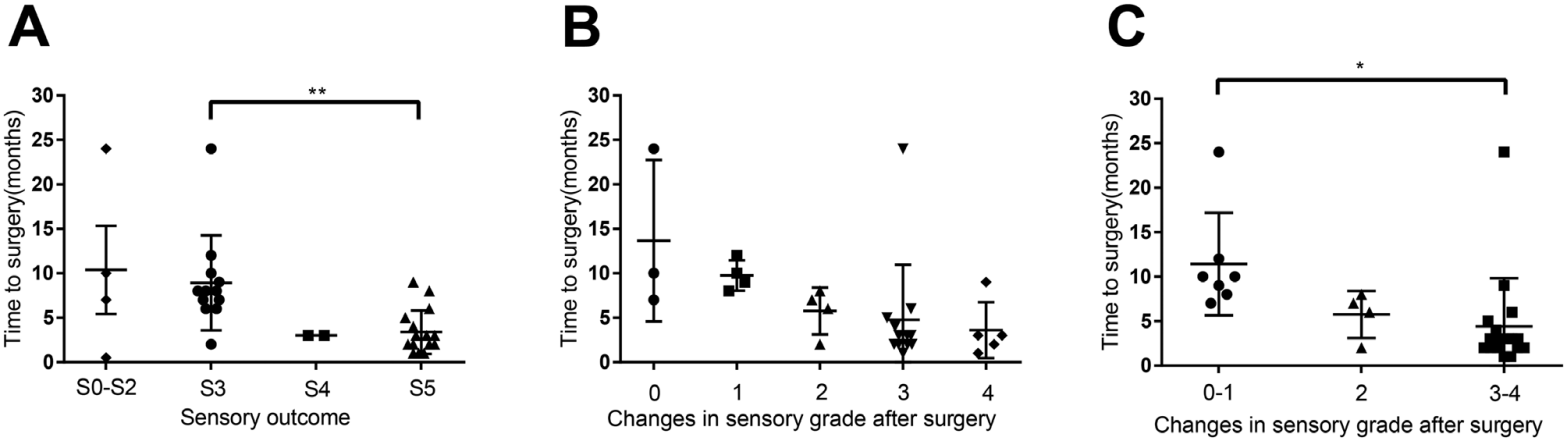
The sensory grade analysis among the three groups. **A**. Relationship between the time from symptom onset to neurolysis and sensory grade (Grade 3 vs. Grade 5). Patients with Grade 5 sensory function (3.38 ± 2.37) had a significantly shorter period from symptom onset to surgical treatment compared to those with Grade 3 (8.92 ± 2.43). *P*=0.002. **B-C**. Time from symptom onset to treatment for patients with different changes in sensory grade. C. Patients with 3 to 4 grade sensory function improvements exhibited shorter intervals (4.41 ± 5.26) than those with 0–1 improvement (11.43 ± 5.34). *P*=0.02. *, *P*<0.05; **, *P*<0.01; error bars represent the standard deviation (SD). *n*=34 for panel **A**, *n*=28 for panels **B-C**

Next, we investigated the recovery of sensory function in the dorsal foot and lateral lower leg following neurolysis. Similar to motor function recovery, we observed that better sensory function recovery was associated with shorter time intervals (Fig. 7A). Assessment of 28 patients with poor or fair sensory function prior to treatment (Table 4) revealed a trend toward sensory function improvement among those with shorter durations between disease onset and neurolysis (Fig. 7B). However, due to limited sample sizes in each group, statistical significance was not established. When combining patients with no improvement and those with only one level of improvement, we found that individuals with shorter time intervals achieved significant sensory function recovery, whereas those with longer intervals experienced limited improvements (Fig. 7C).

**Fig. 8:**
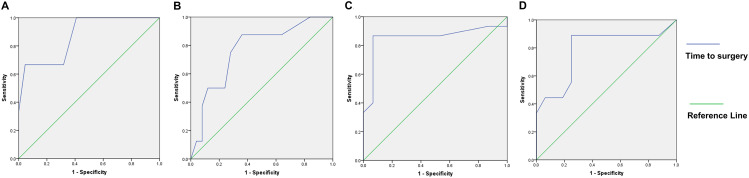
ROC curves for time to surgery. **A**. ROC analysis using M3 foot dorsiflexion recovery as the gold standard, with a best cut-off value of 9.5 months (AUC=0.871, 95% CI=0.661-1.000, *P*=0.04). **B**. ROC analysis using M3 toe dorsiflexion recovery as the gold standard, with a best cut-off value of 5.5 months (AUC=0.768, 95% CI=0.582-0.953, *P*=0.025). **C**. ROC analysis using S4 sensory function recovery as the gold standard, with a best cut-off value of 5.5 months (AUC=0.853, 95% CI=0.693-1.000, *P*=0.001). **D**. ROC analysis using M4 foot dorsiflexion recovery as the gold standard, with a best cut-off value of 5.5 months (AUC=0.785, 95% CI=0.575-0.995, *P*=0.02). *n*=25 for panels **A** and **D**; *n*=33 for panel **B**; *n*=30 for panel **C**. ROC, Receiver Operating Characteristic; AUC, Area Under the ROC Curve

To assess the predictive value of time intervals for neurolysis outcomes, ROC analysis was conducted. Using M3 foot dorsiflexion recovery as the reference standard, the optimal time interval cut-off was 9.5 months, with an AUC area of 0.871 (95% CI = 0.661–1.000, *P* = 0.04; Fig. 8A), indicating that patients undergoing neurolysis within 9.5 months of injury had a good chance of achieving foot dorsiflexion at or above M3. When considering M4 foot dorsiflexion recovery as the reference standard, the optimal cut-off interval was 5.5 months, with an AUC area of 0.785 (95% CI = 0.575–0.995, *P* = 0.02; Fig. 8D). Therefore, for patients aiming for foot dorsiflexion at or above M4, early neurolysis within 5.5 months after injury is advisable. Similarly, when using M3 toe dorsiflexion recovery or S4 sensory function recovery as the reference standards, the optimal cut-off remained at 5.5 months, with AUC areas of 0.768 (95% CI = 0.582–0.953, *P* = 0.025; Fig. 8B) and 0.853 (95% CI = 0.693–1.000, *P* = 0.001; Fig. 8C), respectively. In summary, the best chances of recovering foot dorsiflexion, toe dorsiflexion, and sensory function are associated with neurolysis within 5.5 months after injury. Neurolysis performed between 5.5 and 9.5 months post-injury still allows partial foot dorsiflexion recovery.

## Discussion

The high incidence of CPN injuries presents a significant challenge in selecting the most appropriate treatment. In our study, we explored various treatment options, including conservative treatment, physical therapy, neurolysis, direct suture or nerve graft, and tendon transfer. Each treatment method was selected based on the etiology and severity of the patient’s injury, acknowledging that the right treatment approach can vary significantly depending on these factors. Patients with CPN transection or traction injuries can be considered for tendon transfer, while those with CPN rupture may benefit from nerve graft or tendon transfer and those with CPN compression are often considered for neurolysis [25]. However, in cases of cut traumas, intraoperative exploration has sometimes revealed CPN continuity. When these patients undergo timely surgery, simple neurolysis can lead to significant functional recovery. In a previous study, we did not observe a clear relationship between the causes of injury and postoperative outcomes, which might be attributed to the high incidence of trauma as the primary cause of injury and the varying degrees of injury severity among patients.

The CPN innervates muscles responsible for both foot and toe dorsiflexion. In this study, we aimed to provide a comprehensive assessment of CPN function by including both foot and toe dorsiflexion. While foot dorsiflexion is crucial for gait, toe dorsiflexion, governed by the deep peroneal nerve branch of the CPN, also plays a role in balanced and functional gait, particularly during the swing phase. Our findings revealed that a significant proportion of patients achieved good toe dorsiflexion recovery postoperatively. Specifically, 76% of patients undergoing neurolysis and 60% in the nerve repair group achieved good or fair toe dorsiflexion. This indicates the potential for functional recovery of toe dorsiflexion, which we believe is an important aspect of overall CPN function. The recovery of toe dorsiflexion function showed a significant association with shorter time intervals between symptom onset and surgery, indicating that patients who underwent surgery within shorter time intervals were more likely to achieve improved toe dorsiflexion, highlighting the time-sensitive nature of this aspect of recovery.

Conservative treatment can be effective for some CPN injuries, as spontaneous recovery is possible in certain cases. However, Maalla et al. found that if symptoms do not start to improve within the first month, early surgery within the first few months is advisable, which could otherwise delay or lead to incomplete spontaneous recovery [7]. Some patients with subtle symptoms and no significant findings in EMG may also benefit from surgery [26]. While physical therapy, including electrical stimulation, is a safe clinical approach [27] that can accelerate axon regeneration beyond the site of injury after surgery [28], it should be viewed as a complementary method that requires coordination with surgical intervention. Neurolysis of the CPN generally leads to faster recovery compared to rehabilitation therapy alone [7]. However, not all patients are willing to undergo surgery, and some may not be suitable candidates for neurolysis. Furthermore, there are no well-defined criteria to recommend or avoid neurolysis. Nerve repair has become increasingly effective with advancements in microsurgery techniques, although it tends to yield suboptimal results in patients not treated within 12 months of injury or those requiring grafts longer than 12 cm [2]. Tendon transfer is a common alternative for patients with limited nerve function. However, some patients may be hesitant to undergo tendon transfer, especially when ankle-foot orthoses like shoe dorsiflexion splint inserts can adequately support their daily activities [29].

One significant finding from our study is the independent predictive value of the time elapsed between symptom onset and neurolysis on patient outcomes, which can aid surgeons in making informed decisions regarding surgical interventions. Patients who underwent neurolysis within 5.5 months of their injury achieved substantial recovery in foot/toe dorsiflexion function and sensation. However, those who had surgery between 5.5 and 9.5 months post-injury only experienced partial foot dorsiflexion improvement, and neurolysis was less likely to restore effective function in individuals injured for over 9.5 months. In such cases, alternative options like tendon transfer or nerve repair may be more appropriate. Prior studies have also noted a correlation between the timing of surgery and postoperative recovery [30–32]. Nonetheless, our study offers a more comprehensive and systematic exploration of how CPN neurolysis influences postoperative sensorimotor function recovery, with potential clinical implications. Given the potential for spontaneous recovery in some patients, we cannot definitively attribute the functional improvements observed within shorter time intervals solely to neurolysis. Nevertheless, we can conclude that patients with more favorable motor and sensory functional recoveries tend to have shorter time intervals. For patients with longer intervals, additional treatment modalities may be necessary to facilitate substantial functional recovery. Taken together, our findings provide important insights for clinical decision-making and emphasize the importance of timely surgical intervention.

Neurolysis can achieve favorable outcomes in 80% of patients [2], with reduced functional recovery observed as surgery is delayed [33]. Timely medical attention is crucial, but treatment delays can occur due to patient referral issues [33]. The CPN has a poorer blood supply than the tibial nerve [34, 35], which can lead to irreversible CPN damage with long-term compression, rendering traditional neurolysis less effective.

Some patients may choose observation over neurolysis, as advocated by Rose et al., for a 6-month observation period in peroneal nerve palsy [36]. In our series, all patients (except one) were treated after at least 1 month of observation. However, we found that observation alone did not yield satisfactory results. Despite potential drawbacks, the benefits of surgery outweigh the disadvantages. Currently, a 6–8 cm incision is made at the fibular head, but Ducic et al. recommended a minimally invasive 3 cm approach to reduce surgical trauma [37].

Our results indicated that tendon transfer generally led to better foot dorsiflexion recovery compared to nerve grafting, while nerve grafting was more effective in toe dorsiflexion and sensory function recovery. Giuffre et al. reported a 30% functional recovery rate (*n* = 10) in patients undergoing nerve repair, which is suboptimal [38]. Due to the limited number of nerve repair cases in our study, we refrain from making a definitive conclusion about the efficacy of nerve grafting. Yeap et al. reported that 83% of patients (*n* = 12) who underwent posterior tibial tendon transfer achieved excellent or good outcomes [39], consistent with our findings. Overall, these highlight the need for a tailored approach in treating CPN injuries, considering the specific functional deficits and patient needs.

Nerve repair, also known as nerve graft in our studies, yielded favorable motor recovery in 60% of patients and sensory recovery in 40%. Among our series, four patients had grafts shorter than 6 cm, with three of them achieving good motor function recovery, as reported by Kim et al. [33]. Notably, graft length, rather than the number of cables, significantly influenced the outcomes [31, 40]. Fragility of the nutrient arteries of the CPN is a critical consideration; Lundborg et al. observed complete nerve ischemia with a 15% elongation of nerves [41]. To enhance nerve graft success, it is advisable to minimize graft length, reduce intraoperative nerve stretching, and ensure tension-free anastomosis. However, nerve grafting is inevitably associated with complications, including numbness.

The decision to use the Peroneus brevis tendon in transfers was influenced by its potential to enhance motor function, particularly in cases where nerve repair alone might not suffice. Our findings revealed that 83% of patients who underwent tendon transfer achieved favorable foot dorsiflexion recovery. While toe dorsiflexion function was not fully restored by tendon transfer, one patient exhibited improved toe dorsiflexion postoperatively. This improvement may be attributed to the balancing effect of tendon transfer on foot extension and plantar flexion forces, thereby promoting CPN regeneration. The mean time interval to surgery was 3 years, consistent with the understanding that nerve function may take up to 2 years to recover after nerve repair [42]. However, patients with prolonged foot dorsiflexion dysfunction may develop rigid equinus contracture, potentially leading to permanent deficits in plantarflexion [4]. Early tendon transfer is already widely accepted for ulnar and radial nerve injuries [43, 44], suggesting that it may be a suitable option for patients who do not benefit from neurolysis.

Tendon transfer primarily enhances motor function, while nerve repair offers both motor recovery and sensory improvement. Consequently, combining these complementary procedures can facilitate patient recovery. Milesi’s theory suggests that reinnervation may be impeded by the force imbalance between active plantar flexor muscles and passively stretched denervated foot extensors [45]. Tendon transfer can rectify this imbalance, and simultaneous tendon transfer and nerve graft may enhance rehabilitation according to this theory [40]. Considering the limitations of each surgical method, our study supports the idea of combining tendon transfer and nerve repair to achieve better rehabilitation outcomes. This combined approach, in line with Milesi’s theory, suggests that rebalancing muscle group strength through tendon transfer, alongside nerve repair, can promote more comprehensive patient recovery.

Our research has several limitations that should be considered. First, this was a retrospective study in a single medical center, and the number of patients was limited. Second, data regarding postoperative rehabilitation were not reported in most patient’s reports and could not be analyzed. Third, several patients had incomplete physical examination results, and we did not perform simultaneous tendon transfer and neurolysis or nerve graft and could not evaluate combination procedures. Lastly, we acknowledge that focusing solely on toe dorsiflexion may not fully capture the functional gait outcomes. In future studies, larger sample sizes, more comprehensive data sets and more detailed analysis (i.e., ankle dorsiflexion and its direct impact on gait ability, alongside toe dorsiflexion) would be needed to provide a more holistic understanding of CPN injury recovery and validate these obtained results.

## Conclusions

Our retrospective study on CPN injury therapy demonstrates the effectiveness of surgical treatment in improving clinical outcomes. While some patients may experience spontaneous recovery, our findings suggest that early surgical intervention leads to better outcomes, especially in cases where conservative treatment does not yield significant improvements. Thus, the choice of treatment should be guided not only by the nature of the CPN injury but also by the timing of surgical intervention, which is a crucial factor for motor and sensory function recovery after neurolysis, evidenced by the optimal results achieved when neurolysis was performed within 5.5 months of injury. Neurolysis alone can partially restore foot dorsiflexion function between 5.5 and 9.5 months after injury, but combining it with other procedures yielded the best therapeutic results. For patients who have been injured for more than 9.5 months, neurolysis alone may not be advisable, and in such cases, nerve repair and tendon transfer could be more appropriate options, as nerve repair was found to enhance the recovery of toe dorsiflexion and sensation in the dorsal foot and lateral lower leg. However, patients undergoing nerve repair often experience numbness and occasional pain. Tendon transfer was suitable for patients aiming at improving foot dorsiflexion function to some extent. Taken together, these results could help assist clinicians in selecting appropriate treatment plans tailored to the characteristics and needs of CPN injury patients.

## Data Availability

The datasets used and/or analyzed during the current study are available from the corresponding author on reasonable request.
